# Frequency of delirium and its associated factors among COVID‐19 inpatients in Iran

**DOI:** 10.1111/crj.13609

**Published:** 2023-04-13

**Authors:** Fatemeh Alizadeh Arimi, Mehran Zarghami, Mahmood Moosazadeh, Hossein Mehravaran, Faranak Sedighi, Roya Ghasemian, Forouzan Elyasi

**Affiliations:** ^1^ Student Research Committee, Faculty of Medicine Mazandaran University of Medical Sciences Sari Iran; ^2^ Psychiatry and Behavioral Sciences Research Center, Addiction Institute Mazandaran University of Medical Sciences Sari Iran; ^3^ Department of Psychiatry, Faculty of Medicine Mazandaran University of Medical Sciences Sari Iran; ^4^ Gastrointestinal Cancer Research Center, Non‐communicable Diseases Institute Mazandaran University of Medical Sciences Sari Iran; ^5^ Department of Internal Medicine, Pulmonary and Critical Care Division, Faculty of Medicine Mazandaran University of Medical Sciences Sari Iran; ^6^ Antimicrobial Resistance Research Center, Department of Infectious Diseases Mazandaran University of Medical Sciences Sari Iran; ^7^ Sexual and Reproductive Health Research Center, Addiction Institute Mazandaran University of Medical Sciences Sari Iran

**Keywords:** clinical, COVID‐19, delirium, demographic factors, hospital

## Abstract

**Background and aim:**

Delirium has been presented as the leading cause of sudden change in the mental state of patients with coronavirus disease 2019 (COVID‐19). Given that the delayed diagnosis of such a dysfunction is often associated with excess mortality, it seems essential to devote vastly more attention to this significant clinical characteristic.

**Materials and methods:**

This cross‐sectional study was performed on 309 patients [viz. 259 cases hospitalized in general wards and 50 individuals admitted to the intensive care unit (ICU)]. For this purpose, a Demographic‐Clinical Information Questionnaire, the Confusion Assessment Method (CAM), the Confusion Assessment Method for the ICU (CAM‐ICU), the Richmond Agitation‐Sedation Scale (RASS) and face‐to‐face interviews were completed by a trained senior psychiatry resident. The data analysis was further done with the SPSS Statistics V22.0 software package.

**Results:**

Out of 259 patients admitted to the general wards and 50 cases in the ICU due to COVID‐19, 41 (15.8%) and 11 (22%) individuals were diagnosed with delirium, respectively. As well, a significant relationship was observed between the incidence rate of delirium and age (*p* < 0.001), level of education (*p* < 0.001), hypertension (HTN) (*p* = 0.029), a history of stroke (*p* = 0.025), a history of ischemic heart disease (IHD) (*p* = 0.007), a history of psychiatric disorders, a history of cognitive impairment (*p* < 0.001), use of hypnotic and antipsychotic medications (*p* < 0.001) and a history of substance abuse (*p* = 0.023). Among 52 patients with delirium, only 20 cases had received psychiatric consultation by consultation‐liaison psychiatry service for the possibility of delirium.

**Conclusion:**

In view of the high frequency of delirium among COVID‐19 inpatients, their screening for this important mental state should be a priority in clinical settings.

## Graphical abstract

From 309 hospitalized patients with COVID‐19, 37.8% had delirium. Regarding the high frequency of important complication, further attention should be paid to the mental state of patients with COVID‐19.

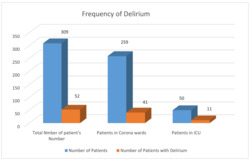

## 1 INTRODUCTION

As an unprecedented global health threat,^1^, ^2^ the coronavirus disease 2019 (COVID‐19) pandemic is affecting all people worldwide, regardless of their age, nationality or socioeconomic status. Nevertheless, older adults are at greater risk of developing respiratory, cardiovascular and brain complications after contracting this infection.^3^ Delirium is also known as the most common diagnosis of the central nervous system (CNS) involvement among patients admitted to the intensive care units (ICUs).^3^, ^4^, ^5^ The incidence rate of this dysfunction in the ICUs varies from 8% to 92%, depending on the patient demographic data, disease severity and the number of mechanically ventilated cases.^6^ Impaired consciousness and confusion might be among the significant symptoms of COVID‐19, even before the fever and cough.^7^

Addressed in previous research, 20%–30% of COVID‐19 patients had suffered from delirium and altered mental status (AMS) during hospitalization, and this value had been as high as 60%–70% in the severe cases of all ages.^8^ In a case series of 58 COVID‐19 patients, where confusion had been determined using the Confusion Assessment Method for the Intensive Care Unit (CAM‐ICU), 84% of the patients had presented neuropsychiatric symptoms, 69% of the individuals had experienced agitation, and 65% of the cases had shown confusion symptoms.^4^ In another report from Wuhan, China, at least 20% of the patients who had died of COVID‐19 had the evidence of encephalopathy.^9^ In the study of 214 COVID‐19 patients, the critically ill cases had shownsymptoms such as impaired consciousness.^3^

In spite of this, it is not yet clear whether neurological syndromes, specifically delirium, are directly caused by the virus entering the CNS or indirectly induced by the systemic inflammatory storms taking place in response to the virus with the explosive release of cytokines, chemokines and other inflammatory mediators.^10^ The mechanisms of this association are probably multifactorial, directly associated with neural invasion and indirectly related to cerebrovascular involvement, hypoxia, high fever, dehydration, inflammation (particularly, cytokine storm syndrome) and use of medications.^8^

The factors that are likely to be connected with delirium in COVID‐19 patients consist of social and epidemiological factors (e.g. social distancing and quarantine and increased occupational workload of healthcare providers), iatrogenic factors (such as the excessive use of sedatives to facilitate mechanical ventilation (MV),^2^ prolonged MV, lengthened immobility, inadequate pain evaluation and alleviation and delayed weaning due to fear of aerosol spread) and psychological ones (viz. fear of death, loneliness, fear of global pandemic, anxiety, future uncertainty, loss of awareness of time and place, lack of religious/spiritual support, hallucination, delusion and sleep disorders).^3^, ^11^ Moreover, it has been suggested that the use of personal protective equipmentand social distancing are likely to have negative effects on patients with delirium and dementia.^12^

There are also some major concerns about the inadequate diagnosis and management of delirium in COVID‐19 patients during this pandemic. Currently, COVID‐19 assessments do not routinely check for delirium and AMS in older adults. This is while the WHO guidelines for patients suspected of COVID‐19 recommend much more attention to the signs of abnormality in the mental state, especially in the elderly population.^8^

Although the association between delirium and COVID‐19 has been highlighted in a number of short reports, case reports and letters to the editors, descriptive and analytical studies in this area have had some limitations, such as no baseline functional assessment, lack of baseline cognitive assessment, failure to report the medications administered^13^ and use of inaccurate tools to measure delirium.^14^ Since the delayed diagnosis of delirium is associated with increased length of stay in the ICUs, excess mortality, lower 6‐month survival rate, longer MV, higher incidence rate of nosocomial pneumonia, augmented risk of delirium recurrence and permanent disability and poor cognitive recovery,^6^ more attention needs to be paid to this area. From this perspective, the present study investigated the frequency of delirium and its relationship with demographic and clinical factors among COVID‐19 inpatients.

## 2 MATERIALS AND METHODS

### 2.1 Research design

This cross‐sectional study was conducted from July 2020 to May 2021 on the confirmed COVID‐19 patients with reverse transcriptase‐polymerase chain reaction assay for SARS‐CoV‐2 RNA that admitted to a teaching hospital, namely, Imam Khomeini Hospital, in Sari, the capital city of Mazandaran province, northern Iran. Subjects were recruited by convenience sampling. Inclusion criteria was confirmed positivity to the SARS‐CoV‐2 virus (with PCR technique).

### 2.2 Sample size

Based on the following formula and on the study of Khan et al,^13^ the appropriate sample size was determined to be 292 people, which was raised to 307 to account for 5% sample loss.

$$n=\frac{Z_{1-\frac{a}{2}}^{2}P\left(1-P\right)}{d^{2}}$$

In this formula, it was assumed that *p* = 0.74, *d* = 0.05, and α/1 = 2.96.

### 2.3 Ethical considerations

This research project was approved by the Ethics Committee of Mazandaran University of Medical Sciences, Sari, Iran (code no. IR.MAZUMS.REC.1399.8251). All the procedures performed in this study, involving human participants, were also in accordance with the ethical standards of the Institutional and/or National Research Committee and the Declaration of Helsinki (DoH) 1964 and its later amendments or comparable ethical guidelines. Informed consent was further obtained from the patients. Consent from the legal guardian was also acquired for the cases with the inability to do so.

### 2.4 Implementation procedure

Upon identifying the potential participants, the interviewer, as a trained senior psychiatry resident, visited the patients. After obtaining their consent, the medical records were studied to collect the demographic and clinical information, and then the questionnaires were read and completed. The patients were also examined at the time of discharge in order to record their clinical conditions.

### 2.5 Research tools

The assessment tools utilized in this study were a Demographic‐Clinical Information Questionnaire, the Confusion Assessment Method (CAM), the CAM‐ICU and the Richmond Agitation‐Sedation Scale (RASS).

### 2.6 Demographic‐Clinical Information Questionnaire

The information collected by the Demographic‐Clinical Information Questionnaire included age, gender, ward type, marital status, place of residence, level of education, occupation, body mass index (BMI), basic mobility status before admission, pre‐admission condition, contact with family during hospitalization, a history of smoking, a history of substance abuse, a history of previous medical condition, manner of being diagnosed with COVID‐19, a history of psychiatric disorders, symptoms on admission, symptoms during hospitalization, medications received during hospitalization, counseling with different subspecialties all through hospitalization, delirium diagnosis in psychiatric consultations, procedures carried out for the period of hospitalization, respiratory condition, intubation duration and treatment outcome.

### 2.7 CAM

Providing a diagnostic tool for detecting delirium, the CAM measures the presence, severity and fluctuation of nine symptoms of delirium, including acute onset and fluctuating course, inattention, disorganized thinking, altered level of consciousness, disorientation, memory impairment, perceptual disturbance, psychomotor agitation or retardation and altered sleep–wake cycle. In this tool, the data are collected by open‐ended items, which means the patient must be able to speak and answer the questions.^15^, ^16^ The delirium diagnosis is also based on four core features of this condition, namely, (1) acute onset and fluctuating course, (2) inattention, (3) disorganized thinking and (4) altered level of consciousness.^15^ The interviewers accordingly need to rate each feature based on daily observations.^17^ To rate the severity of delirium, they must give each feature—except fluctuation—a score of 0 for no symptoms, 1 for the mild symptoms or 2 for the severe ones and then give fluctuation a score of 0 or 1 based on whether the symptoms fluctuate. Summing up these scores then provides the total delirium severity score, which varies from 0 to 7, with higher scores indicating more severe delirium. This assessment tool has been widely exploited in clinical practice as well as previous research,^15^ with a sensitivity of 94%–100%, a specificity of 90%–95%, a positive predictive value of 91%–94% and a negative predictive value of 90%–100%. Moreover, its high reliability with the Cronbach's alpha coefficient of 0.81–1 has been already confirmed.^15^, ^16^

### 2.8 CAM‐ICU

This assessment tool was originally designed and validated by Ely et al^18^ to eliminate the major shortcoming of the CAM, requiring the patients to be able to speak, which seems impossible in many instances (e.g. in cases with intubation). In this respect, delirium is diagnosed based on four core features, namely, (1) acute onset and fluctuating course, (2) inattention, (3) altered level of consciousness and (4) disorganized thinking. Delirium diagnosis is further done with reference to the presence of the first and second features and either one of the third or fourth features.^16^, ^19^

In a study conducted in Iran on the Persian version of the CAM‐ICU, it had been established to have a sensitivity and specificity of respectively 0.71 and 0.97 in the acute onset–fluctuating course dimension and 0.69 and 0.94 in the inattention dimension. As well, the values of 0.74 and 0.97 in the altered level of consciousness dimension and 0.71 and 0.97 in the disorganized thinking dimension had been reported in this sense. This assessment tool also had good reliability with the Cronbach's alpha coefficient of 0.87.^20^

### 2.9 RASS

Developed by Sessler et al, the RASS is a valid and reliable tool for assessing the sedation status of patients admitted to the ICUs.^21^ It is a 10‐point scale with scores ranging from −5 (lowest) to +4 (highest), which are categorized at three levels.^22^, ^23^ The patients with the RASS score of −4 (i.e. no response to voice but movement or eye opening to physical stimuli) or −5 (viz. no response to voice or physical stimuli) are not eligible for the CAM‐ICU implementation. The patients with the RASS score of −3 or higher and positive CAM‐ICU results are thus classified as delirious.^24^ In a study assessing the validity and reliability of the RASS in the ICUs, the results had found it to be highly correlated with the nurses' Visual Analogue Scalewith the Spearman's correlation coefficient of 0.81 and highly reliable with the Cohen's kappa coefficient of 0.82.^25^ In a survey in Iran, the content validity of the Persian version of this assessment tool had been further established, and its inter‐rater reliability had been already settled with the intracluster correlation coefficient (ICC) of 0.95.^24^

### 2.10 Statistical analysis

To perform the statistical analyses, the data were imported into the SPSSV22.0 software. For the demographic variables, these analyses were fulfilled using descriptive statistics, including mean and standard deviationfor the quantitative data and frequency and percentage for the qualitative ones. The normality of the data was further assessed using the Kolmogorov–Smirnov test. Logistic regression was also utilized to identify the demographic and clinical factors associated with the incidence rate of delirium in COVID‐19 patients. In all analyses, the statistical significance was *p* < 0.05.

## 3 RESULTS

This cross‐sectional study was performed on 309 COVID‐19 inpatients, including 259 cases (83.81%) hospitalized in the general wards and 50 individuals (16.19%) admitted to the ICU.

### 3.1 Demographic characteristics

The mean age of the patients was 58.35 ± 15.60. Most of the study participants were also male (*n* = 159, 55.8%), married (*n* = 256, 76.9%) and illiterate (*n* = 110, 65.4%). In terms of contact with family during hospitalization, the group with the highest frequency was ‘face‐to‐face contact with one person’ (53.7%) (Table 1).

**TABLE 1:** Demographic characteristics of patients.

Variable	Frequency *N*	Percentage %
Gender		
Female	150	48.5
Male	159	51.5
Marital status		
Single	53	17.2
Married	256	82.8
Level of education		
Illiterate	110	35.6
Elementary education	86	27.8
High school diploma	68	22
Bachelor's degree and higher	45	14.6
Level of contact with family during hospitalization		
No contact	52	16.8
Face‐to‐face contact with one person	166	53.7
Contact via voice or video calls	51	16.5
Unknown	40	12.9
Age range		
Less than 60 years	157	50.8
60 years and older	152	49.2

### 3.2 Clinical conditions

**FIGURE 1:**
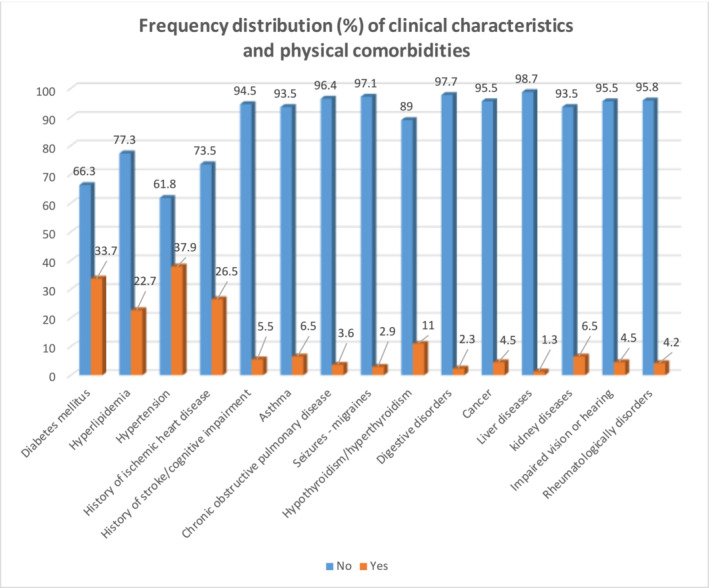
Percentage of different comorbidities in COVID‐19 patients.

**FIGURE 2:**
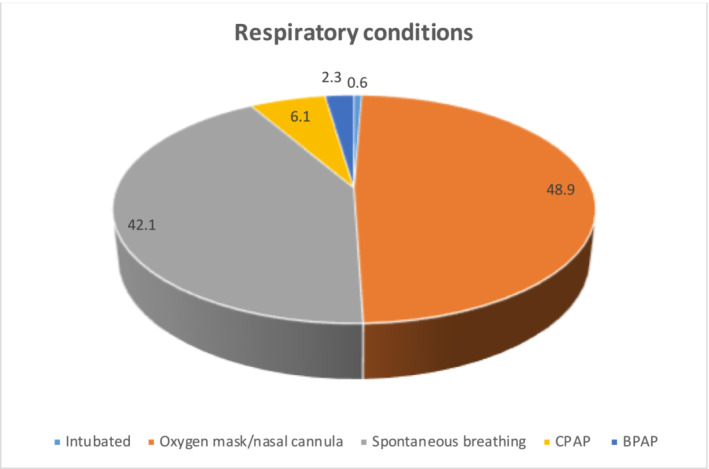
Respiratory conditions in COVID‐19 patients.BPAP, Bi‐level positive airway pressure; CPAP, Continuous positive airway pressure.

The mean BMI was 28.06 ± 4.79. The majority of the patients (*n* = 89, 39.6%) had the BMI between 25 and 29.9. Out of all patients, 130 cases (42.1%) had spontaneous breathing, 151 individuals (48.9%) had been given oxygen mask or nasal cannula, and 19 participants (6.1%) were under continuous positive airway pressure. As well, seven cases (2.3%) were undergoing bi‐level positive airway pressure, and two patients had been intubated (Figures 1 and 2).

### 3.3 Psychiatric disorders and substance abuse comorbidities

Among all participants, 35 cases (11.3%) had a history of psychiatric disorders. Besides, 41 patients (13.1%) had a history of substance abuse. Out of the patients with a history of substance use, six individuals (1.9%) were identified with the use of multiple substances. The most common category of substances was also opioids with the frequency of 5.8% (Table 2).

**TABLE 2:** Frequency distribution of psychiatric disorder and substance abuse comorbidities.

Variable		Frequency *N*	Percentage %
History of psychiatric disorders	Anxiety disorders	10	3.2
	Obsessive–compulsive disorder	1	0.3
	Psychosis/bipolar disorder	1	0.3
	Unipolar mood disorder	11	3.6
	Delirium	1	0.3
	Other psychiatric disorders	11	3.6
	Total	35	11.3
No history of psychiatric disorders	N/A	274	88.7
History of substance abuse	Tobacco	10	3.2
	Alcohol	1	0.3
	Amphetamine	2	0.6
	Opioids	18	5.8
	Methadone	4	1.3
	Multiple drugs	6	1.9
	Total	41	13.1
No history of substance abuse	N/A	268	86.7

### 3.4 Incidence rate of delirium

Among 259 patients hospitalized in the general wards and 50 cases admitted to the ICU, 41 (15.8%) and 11 (22%) individuals had delirium, respectively. In this respect, there was no statistically significant difference between those in the general wards and the ICU in terms of the frequency of delirium (*p* = 0.303). Moreover, 41 patients (15.8%) out of 257 cases hospitalized in the general wards for COVID‐19 and 11 patients (22%) out of 50 individuals in the ICU were suffering from delirium, diagnosed using the CAM and the CAM‐ICU, respectively. It was also observed that a psychiatric consultation with the possibility of delirium had been requested by the medical service for only 20 patients among 52 cases. According to the CAM‐ICU outcomes, 11 patients had delirium. Likewise, two cases (18.2%) had been affected with hyperactive delirium, eight individuals (72.7%) had hypoactive delirium, and one subject (9.1%) had experienced mixed delirium. With reference to the CAM results, 41 cases (100%) with delirium had acute onset. In item 1 in the CAM, related to inattention, 26 patients (63.4%) had mild inattention, 12 cases (29.3%) had been subjected to severe inattention, and one person (2.4%) had no inattention. In relation to item 2 of inattention, 20 patients (48.8%) had inattention. In keeping with the CAM, 26 patients (63.4%) had disorganized thinking, 34 cases (82.9%) had been affected with disorientation, 35 individuals (85.4%) had memory impairment, and three people (7.3%) had undergone perceptual disturbance. Likewise, 13 patients (31.7%) were suffering from no psychomotor agitation, 20 patients (48.8%) had psychomotor retardation, and 35 patients (85.4%) had experienced altered sleep–wake cycle.

### 3.5 Frequency distribution of delirium in terms of demographic‐clinical characteristics and physical comorbidities

The mean age of the patients with delirium was significantly higher than that in the cases without this condition (*p* = 0.000). Moreover, delirium was significantly more frequent among the patients aged 60 or older (*p* < 0.001). There was also no statistically significant difference between the male and female patients with regard to the incidence rate of delirium (*p* = 0.544). Delirium was significantly more frequent among the illiterate patients (*p* < 0.001). Besides, there was no statistically significant difference between the single and married patients in respect of delirium frequency (*p* = 0.228). Delirium was significantly more frequent among the patients with hypertension (HTN) (*p* = 0.029), cardiovascular diseasesor heart attack (*p* = 0.007), a history of stroke (*p* = 0.025) and cognitive disorders (*p* < 0.001) than those without these conditions. There was also no correlation between BMI and the incidence rate of delirium (Table 3).

**TABLE 3:** Distribution of delirium in terms of demographic‐clinical characteristics and physical comorbidities.

Variable	Delirium		*P*‐value
	Yes^a^	No^a^	
Gender			0.544^b^
Female	23 (44.2)	127 (49.4)	
Male	29 (55.8)	130 (50.6)	
Marital status			0.228^b^
Single	12 (23.1)	41 (16)	
Married	40 (76.9)	216 (84)	
Level of education			0.000^c^
Illiterate	34 (65.4)	76 (29.6)	
Elementary education	13 (25)	73 (28.4)	
High school diploma	5 (9.6)	63 (24.5)	
Bachelor's degree and higher	0 (0)	45 (17.5)	
Level of contact with family during hospitalization			0.057^c^
No contact	11 (21.2)	41 (16)	
Face‐to‐face contact with one person	32 (61.5)	134 (52.1)	
Contact via voice or video calls	2 (3.8)	49 (19.1)	
Unknown	7 (13.5)	33 (12.8)	
Age range			0.000^c^
Less than 60 years	8 (15.4)	149 (58)	
60 years and older	44 (84.6)	108 (42)	
Age	52 (70.29 ± 10.96)^d^	257 (55.94 ± 15.30)^d^	0.000^c^
BMI			0.442^c^
<25	11 (42.3)	56 (28.1)	
25–29.9	8 (30.8)	81 (40.7)	
30–34.9	6 (23.1)	45 (22.6)	
>35	1 (3.8)	17 (8.5)	
Diabetes mellitus			0.107^b^
Yes	23 (44.2)	81 (31.5)	
No	29 (55.8)	176 (68.5)	
Hyperlipidemia			0.717^b^
Yes	13 (25)	57 (22.2)	
No	39 (75)	200 (77.8)	
HTN			0.029^b^
Yes	24 (46.2)	93 (36.2)	
No	28 (53.8)	164 (63.8)	
History of IHD			0.007^b^
Yes	20 (38.5)	62 (24.1)	
No	32 (61.5)	195 (75.9)	
History of stroke/cognitive impairment			0.000^b^
Yes	10 (19.2)	7 (2.7)	
No	42 (80.8)	250 (97.3)	
Asthma			0.399^b^
Yes	2 (3.8)	18 (7)	
No	50 (96.2)	239 (93)	
Chronic obstructive pulmonary disease			0.903^b^
Yes	2 (3.8)	9 (3.5)	
No	50 (96.2)	248 (96.5)	
Convulsions‐migraines			0.179^b^
Yes	3 (5.8)	6 (2.3)	
No	49 (94.2)	251 (97.7)	
Hypothyroidism/hyperthyroidism			0.230^b^
Yes	3 (5.8)	31 (12.1)	
No	49 (94.2)	226 (87.9)	
Digestive disorders			0.856^b^
Yes	1 (1.9)	6 (2.3)	
No	51 (98.1)	251 (97.7)	
Cancer			0.053^b^
Yes	5 (9.6)	9 (3.5)	
No	47 (90.4)	248 (96.5)	
Liver diseases			0.660^b^
Yes	1 (1.9)	3 (1.2)	
No	51 (98.1)	254 (98.8)	
Kidney diseases			0.104^b^
Yes	6 (11.5)	14 (5.4)	
No	46 (88.5)	243 (94.6)	
Impaired vision or hearing			0.638^b^
Yes	3 (5.8)	11 (4.3)	
No	49 (94.2)	246 (95.7)	
Rheumatic diseases			0.368^b^
Yes	1 (1.9)	12 (4.7)	
No	51 (98.1)	245 (95.3)	
BMI	26 (26.65 ± 4.86)^d^	199 (28.24 ± 4.7)^d^	0.112^e^

^a^ Frequency (percentage).

^b^ Inter‐group comparison with Fisher's exact test.

^c^ Inter‐group comparison with chi‐square test.

^d^ Frequency (mean ± SD).

^e^ Inter‐group comparison with independent‐samples *t*‐test.

* Significance level = *p* < 0.05.

### 3.6 Frequency distribution of delirium by a history of psychiatric disorders

The chi‐square test results demonstrated a significantly higher frequency of delirium in the patients with a history of psychiatric disorders than those with no history of such conditions (*p* = 0.014). A significant relationship was further detected between the use of hypnotic and antipsychotic medications and the incidence rate of delirium (*p* < 0.001). However, no significant relationship was observed between the frequency of delirium and the use of other medications (*p* < 0.05). There was also a significant relationship between substance abuse and the incidence rate of delirium (*p* = 0.023). A significant relationship was additionally spotted between the frequency of delirium and weakness and lethargy (*p* = 0.021), muscle pain (*p* < 0.001) and decreased level of consciousness on admission (*p* < 0.001). Delirium was significantly more frequent in the patients with muscle pain (*p* = 0.049) and lower level of consciousness during hospitalization (*p* < 0.001). Moreover, there was a significant relationship between the incidence rate of delirium and the use of meropenem‐imipenem (*p* = 0.002) and ceftriaxone all through hospitalization (*p* = 0.004). Overall, the cases receiving more medications for the duration of hospitalization were more likely to develop delirium (*p* = 0.011) (Table 4).

**TABLE 4:** Distribution of delirium in terms of medication regimen and number of drugs administered during hospitalization.

Variable	Delirium		*P*‐value
	Positive^a^	Negative^a^	
Hydroxychloroquine			0.101^b^
Yes	11 (21.2)	87 (33.9)	
No	41 (78.8)	170 (66.1)	
Atazanavir			0.127^b^
Yes	30 (57.7)	116 (45.1)	
No	22 (42.3)	141 (54.9)	
ReciGen (interferon beta‐1a)			0.364^b^
Yes	3 (5.8)	25 (9.7)	
No	49 (94.2)	232 (90.3)	
Enoxaparin‐heparin			0.053^b^
Yes	33 (63.5)	198 (77)	
No	19 (36.5)	59 (23)	
Melatonin			0.053^b^
Yes	5 (9.6)	9 (3.5)	
No	47 (90.4)	248 (96.5)	
Corticosteroids			0.073^b^
Yes	38 (73.1)	216 (84)	
No	14 (26.9)	41 (16)	
Zinc–selenium–vitamin C			0.480^b^
Yes	37 (71.2)	196 (76.3)	
No	15 (28.8)	61 (23.7)	
Azithromycin			0.477^b^
Yes	10 (19.2)	63 (24.5)	
No	42 (80.8)	194 (75.5)	
Meropenem‐imipenem			0.002^b^
Yes	20 (38.5)	46 (17.9)	
No	32 (61.5)	211 (82.1)	
Vancomycin			0.122^b^
Yes	11 (21.2)	32 (12.5)	
No	41 (78.8)	225 (87.5)	
Ceftriaxone			0.004^b^
Yes	18 (34.6)	147 (57.2)	
No	34 (65.4)	110 (42.8)	
Naproxen			0.799^b^
Yes	4 (7.7)	24 (9.3)	
No	48 (92.3)	233 (90.7)	
Famotidine			0.752^b^
Yes	32 (61.5)	166 (64.6)	
No	20 (38.5)	91 (35.4)	
Paracetamol (Apotel®)			0.095^b^
Yes	42 (80.8)	176 (68.5)	
No	10 (19.2)	81 (31.5)	
Remdesivir			0.137^b^
Yes	11 (21.2)	84 (32.7)	
No	41 (78.8)	173 (67.3)	
Favipiravir			0.112^b^
Yes	0 (0)	12 (4.7)	
No	52 (100)	245 (95.3)	
Total number of medications	52 (15.46 ± 4.82)^c^	257 (13.86 ± 4.00)^c^	0.011^d^
Oral medications	52 (8.81 ± 3.40)^c^	257 (7.89 ± 3.15)^c^	0.059^d^
Injectable medications	52 (6.21 ± 2.23)^c^	257 (5.73 ± 1.81)^c^	0.093^d^

^a^ Frequency (percentage).

^b^ Inter‐group comparison with Fisher's exact test.

^c^ Frequency (mean ± SD).

^d^ Inter‐group comparison with independent‐samples *t*‐test.

* Significance level = *p* < 0.05.

### 3.7 Frequency distribution of delirium as for consultation with other specialty during hospitalization

The patients who had received infectious disease or psychiatric consultation were more likely to have delirium (*p* = 0.013 and *p* < 0.001, respectively). Out of 52 patients with delirium, only 20 cases (26%) had received psychiatric consultation by a prior consultation‐liaison psychiatry service and appropriate delirium treatment.

### 3.8 Frequency distribution of delirium in relation to procedures performed on patients

Among 309 patients, 178 cases had received one diagnostic‐therapeutic intervention, and 131 participants had taken more than one diagnostic‐therapeutic intervention. Out of 178 patients with one intervention, seven people (13.5%) had delirium. But, among 131 patients with two or more interventions, 45 cases (86.5%) had experienced delirium. Moreover, delirium was significantly more frequent in the patients with more than one diagnostic‐therapeutic intervention than those with one intervention (*p* < 0.001).

### 3.9 Frequency distribution of delirium in terms of respiratory condition, oxygen saturation level and lung imaging findings

A significant relationship was detected between the incidence rate of delirium and respiratory condition (*p* = 0.004) and ejection fraction (EF) (*p* = 0.040). Although the patients who had a lower oxygen saturation level during hospitalization were more likely to have delirium, this relationship was not statistically significant (*p* = 0.099).

### 3.10 Frequency distribution of delirium with regard to prognosis‐affecting laboratory findings on admission

No significant relationship was found between the laboratory findings that could affect prognosis, including C‐reactive protein, ferritin, D‐dimer, lactate dehydrogenase (LDH) and interleukin 6and the incidence rate of delirium in the patients (*p* < 0.05). Delirium was also more frequent among the cases with more white white blood cellcount (*p* = 0.021), higher prothrombin time(*p* = 0.098), aspartate transaminase(*p* = 0.057), urea (*p* < 0.001), creatinine (*p* = 0.019), erythrocyte sedimentation rate (ESR) (*p* = 0.005), creatine phosphokinase(*p* = 0.073) and direct bilirubin levels (*p* = 0.096), as well as lower albumin (*p* = 0.002) and lymphocyte (*p* = 0.067).

### 3.11 Logistic regression models for predictors of frequency of delirium

Delirium was about 70% less frequent in the participants with elementary education than the illiterate cases. After controlling the effect of the confounding variables, delirium was 82% more frequent in the cases with a history of stroke/cognitive impairment than those with no history of these conditions. It was further reported that delirium increased by 1.028 units for each unit rise in ESR, an association that was on the border of statistical significance (*p* = 0.052) (Table 5).

**TABLE 5:** Regression model based on study variables.

Variable		Univariate			Multivariate		
		OR	95% CI	*P*‐value	OR	95% CI	*P*‐value
Total number of medications		1.09	1.01–1.17	0.13	1.008	0.87–1.15	0.910
Erythrocyte Sedimentation Rate on admission		1.017	1.00–1.03	0.006	1.028	1.0–1.05	0.052
Number of procedures performed		12.782	5.53–29.5	0.000	14.37	2.42–85.3	0.003
Ejection fraction	<40%	Ref.	Ref.	0.034	Ref.	Ref.	0.433
	40–50%	1.286	0.305–0.426	0.732	2.103	0.25–17.6	0.494
	>50%	0.327	0.069–1.54	0.159	0.838	0.089–7.92	0.877
Age range		7.588	3.43–16.7	0.000	2.339	0.31–17.6	0.410
History of psychiatric disorders		2.605	1.18–5.72	0.017	0.988	0.089–10.9	0.992
Use of hypnotic and antipsychotic medications		3.453	1.68–7.09	0.001	1.398	0.12–16.09	0.788
History of stroke/cognitive impairment		8.503	3.06–23.57	0.000	1.817	0.082–40.1	0.705
Level of education	Illiterate	Ref.	Ref.	0.002	Ref.	Ref.	0.587
	Elementary education	0.398	0.19–0.81	0.012	0.320	0.059–1.73	0.186
	High school diploma	0.177	0.06–0.48	0.001	0.450	0.022–9.33	0.605
	Bachelor's degree and higher	0.000	0.000	0.997	0.000	0.00	0.998

## 4 DISCUSSION

This study aimed to investigate the frequency of delirium and its relationship with the demographic‐clinical factors in COVID‐19 inpatients at a teaching hospital in northern Iran. In this respect, 16.82% of the cases out of 309 eligible participants admitted to the hospital due to COVID‐19 had delirium. As well, 41 out of 259 patients hospitalized in the general wards (15.8%), and 11 cases out of the 50 patients in the ICU (22%) were suffering from delirium. In a retrospective study by García‐Grimshaw et al on 1017 COVID‐19 patients aged 18 and older who had been hospitalized in Mexico City, the frequency of delirium was 166 cases (16.3%),^26^ which was very close to the value observed in the present study, even though a larger sample size had been surveyed in García‐Grimshaw et al.'s research. Since the evaluations in their study had been performed by both doctors and nurses, the accuracy of their findings might have been affected by the multiplicity of evaluators. In a cohort study, Pun et al had examined the incidence rate of delirium in 2088 COVID‐19 patients with severe symptoms admitted to the ICUs in 16 different countries. Excluding the patients younger than 18 and those with a history of liver disease, blindness and deafness, suicidal ideation and neurodegenerative diseases, according to the Scales for the Assessment of Positive Symptoms II (SAPS II) and the RASS, they had correspondingly found that 1147 patients (54.9%) had delirium with the median RASS score of −4 and the delirium duration of 2–6 days (the median of 3 days).^27^ The difference between the frequency values in their survey and those in the present study can be attributed to the fact that they had merely reflected on the ICU patients, which could naturally have more severe symptoms. Furthermore, the participants in their study were older, which could put them at higher risk of delirium. While there was a much larger sample size in Pun et al than that in the present study, which can strengthen the reliability of their results, many physical conditions that could be an underlying cause or trigger for delirium had been disregarded.

In Italy, a multicenter observational research was conducted on 80 patients with confirmed COVID‐19 and concurrent delirium. The type of this study conducted by Giovanni Martinotti et al was a cohort type, which is different from the present study in nature. In addition, despite the fact that the aim of the study was to determine the incidence of delirium and other neuropsychiatric symptoms, the incidence was not determined. While in the present study, the frequency of delirium in 309 patients with confirmed COVID‐19 was determined. In addition, the measurement tool of that study for delirium was the 4 ‘A's Test, which only takes a few minutes, but in the present study, more reliable tools were used.^28^ Moreover, another retrospective study was conducted in elderly patients with COVID‐19 in London, and the frequency of delirium in that population was 23.9%. In the limitations of their study, the authors mentioned that the retrospective nature makes the data interpretation to be done with caution. In addition, that study was conducted only on the elderly population, who are at risk of delirium, while the present study was conducted in a wider age rangeand with a study conducted on different ages, the relationship between age and delirium can be better understood so it answers the question of whether age is a risk factor for delirium in COVID‐19 patients, like other etiologies or not.^29^

The mean age of the patients in the present study was 58.35 ± 15.60. The majority of the cases were male (*n* = 159, 55.8%), married (*n* = 256, 76.9%) and illiterate (*n* = 110, 65.4%). A significant relationship was further detected between the incidence rate of delirium during hospitalization and age, level of education, HTN, a history of stroke, a history of psychiatric disorders, use of hypnotic and antipsychotic medications, a history of substance abuse, muscle pain and decreased level of consciousness on admission. There was also a significant relationship between the frequency of delirium and the use of ceftriaxone as well as meropenem‐imipenem all through hospitalization, number of diagnostic‐therapeutic procedures performed and respiratory EF conditions in the patients. The positive relationship observed in this study between illiteracy and the incidence of delirium was probably due to the advanced age of illiterate Iranians, which could make age a confounding factor for the incidence rate of delirium among the illiterate patients. Thus, the role of age in the higher frequency of delirium in illiterate patients should not be overlooked. In the study by Ticinesi et al on 582 COVID‐19 patients in Italy, 94 cases (11%) who had developed delirium were significantly older than other patients (*p* ˂ 0.001) and also had more neuropsychological comorbidities and worse respiratory conditions. The frequency of delirium was also associated with the use of antipsychotic medications (*p* = 0.025), serum urea and LHD levels. In their study, mortality rate had multiplied with delirium irrespective of age and respiratory conditions.^30^ Although antipsychotic medications are part of the drug regimen for delirium, there was a positive correlation between the use of such medications and the incidence rate of delirium in the present study, which could be related to the multitude of drugs taken by the patients or their underlying psychiatric illnesses.

In an observational study by Khan et al on the incidence, duration and severity of delirium in 268 critically ill COVID‐19 patients with the mean age of 58.4, who had been admitted to the ICUs of two major academic centers in Indiana, USA,^13^ no significant difference had been reported between the patients who were positive and negative for delirium in terms of mortality, gender, race or comorbidity. However, there were statistically significant differences in terms of the RASS score, the Glasgow Coma Scalerange, the presence of acute physiological shock and the chronic health assessment outcomes.^13^ While the significant relationship observed between the frequency of delirium and older age and sedative use in Khan et al.'s study was consistent with the findings in the present study, the same was not true for a history of substance abuse and HTN. Considering that the assessment of the delirium outcomes in this study was limited to the hospitalization length, it was possible to obtain more accurate results from longer follow‐ups, because the risk of delirium‐related morbidity and mortality could sometimes persist up to 1 year after recovery.^13^ In a study by Kotfis et al, they observed delirium in 70%–75% of the ICU patients with COVID‐19 undergoing MV and found it to be associated with longer hospital stays and higher mortality rates.^31^ Delirium had also lasted for 5 days on average. Moreover, MV was significantly associated with the likelihood of delirium even after adjustment for sedative use. The high mortality rate in their survey compared with the outcomes in the present study could be thus related to the poor physical condition of the patients admitted to the ICU. Besides, the study findings here suggested that performing a greater number of procedures on the patients could redouble the risk of delirium, which might explain the higher incidence rate of this condition in the patients in Kotfis et al, undergoing MV.

In a multicenter cohort study conducted by Pun et al on all patients with acute respiratory syndrome, aged over 18, admitted to the ICUs due to the COVID‐19 infection, sedative infusion had been very common during MV. Out of 2088 patients examined, 1337 cases (64%) had received benzodiazepines for an average of 7 days and 1481 individuals (70.9%) had taken propofol for an average of 1 week. They had further found that MV, use of physical restraint and administration of benzodiazepines, opioids, vasopressors and antipsychotic drugs could be associated with a higher risk of delirium in the following day, and even personal or virtual family visits could be associated with a lower risk of delirium. Moreover, they had reported that older age, higher SAPS II scores, the male gender, smoking or alcohol use, taking vasopressors and aggressive MV on the first day of admission could be independently associated with shorter survival rates and longer delirium and coma. Out of 2088 patients, 601 cases had also died within 28 days of admission, predominantly in the ICUs. Overall, the findings had shown the common occurrence of prolonged acute brain dysfunction in critically ill COVID‐19 patients. Furthermore, benzodiazepine use and lack of family visits had been identified as the modifiable risk factors for delirium.^27^

On the contrary, the findings in the present study showed that contact with family members during hospitalization made no significant difference in the incidence rate of delirium. Considering the exclusion of the potential impacts of blindness and deafness as well as a history of physical and psychiatric problems in the patients on the development and exacerbation of delirium in Pun et al, the study results here did not validate a statistically significant difference in this regard that might be related to the small sample size.

One of the limitations in this study was its cross‐sectional design. As well, the severity or duration of delirium during hospitalization was not recorded, which could be important because there is evidence that COVID‐19 patients experience longer periods of delirium than non‐COVID‐19 cases.^11^ Prospective studies can thus help to address this shortcoming.

## 5 CONCLUSION

With regard to the high frequency of delirium in COVID‐19 inpatients, it is recommended to routinely screen such cases for this serious change in the mental state with appropriate assessment tools in order to ensure early diagnosis followed by timely adoption of appropriate measures to manage it.

## AUTHOR CONTRIBUTIONS

This article has been extracted from psychiatric specialty dissertation of Fatemeh Alizadeh Arimi. Fatemeh Alizadeh Arimi participated in study design, data collection and interpretation and revision and interpretation and revision of manuscript. Forouzan Elyasi participated in study design, interpreted the findings and drafted and revised the manuscript. Faranak Sedighi participated in collecting the data. Roya Ghasemian and Hossein Mehravaran participated in clinical management of the patients. Mahmood Moosazadeh performed the statistical analysis. Mehran Zarghami participated in study design, re‐evaluated the data, interpreted the findings and revised the manuscript. All authors read and approved the final manuscript.

## CONFLICT OF INTEREST STATEMENT

The authors declared no competing interests in this study.

## ETHICS STATEMENT

This research project was approved by the Ethics Committee of Mazandaran university of Medical sciences (IR.MAZUMS.REC.1399.8251), Sari, Iran. Informed consents were obtained from all participants, and they were ensured that their identity would be kept anonymous throughout the study.

## Data Availability

The datasets generated during and/or analysed during the current study are available from the corresponding author on reasonable request.
